# [2,6-Bis(di-*tert*-butyl­phosphinometh­yl)phenyl-κ^3^ *P*,*C* ^1^,*P*′](trifluoro­acetato)palladium(II)

**DOI:** 10.1107/S1600536810017861

**Published:** 2010-05-19

**Authors:** Magnus T. Johnson, Mario Cetina, Kari Rissanen, Ola F. Wendt

**Affiliations:** a Organic Chemistry, Department of Chemistry, Lund University, PO Box 124, S-221 00 Lund, Sweden; b Department of Chemistry, Nanoscience Centre, University of Jyväskylä, PO Box 35, FIN-40014 Jyväskylä, Finland

## Abstract

The Pd^II^ atom in the title compound, [Pd(C_2_F_3_O_2_)(C_24_H_43_P_2_)], adopts a distorted square-planar geometry with the P atoms in a *trans* arrangement, forming two five-membered chelate rings. Four intra­molecular C—H⋯O hydrogen bonds occur. The crystal packing reveals one weak inter­molecular C—H⋯O hydrogen bond, which self-assembles the mol­ecules into infinite chains parallel to the *b* axis.

## Related literature

For synthetic procedures, see: van der Boom *et al.* (1998 ▶); Johansson *et al.* (2005 ▶). For a similar complex with two six-membered rings in the framework, see: Ohff *et al.* (1997 ▶). For a crystallographic analysis of related complexes, see: Johansson *et al.* (2007 ▶). For similar complexes with ruthenium and nickel, see: Zhang *et al.* (2005 ▶) and Boro *et al.* (2008 ▶), respectively. For reactivity studies of the title compound, see: Johansson & Wendt (2007 ▶).
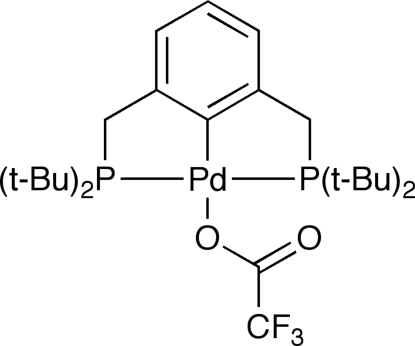


## Experimental

### 

#### Crystal data

[Pd(C_2_F_3_O_2_)(C_24_H_43_P_2_)]
*M* *_r_* = 612.94
Orthorhombic, 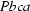

*a* = 11.1239 (1) Å
*b* = 15.7484 (2) Å
*c* = 32.4400 (4) Å
*V* = 5682.96 (11) Å^3^
*Z* = 8
Mo *K*α radiation
μ = 0.81 mm^−1^
*T* = 123 K
0.2 × 0.1 × 0.1 mm

#### Data collection

Bruker–Nonius Kappa APEXII diffractometer
Absorption correction: multi-scan (*SADABS*; Sheldrick, 2008*a* ▶) *T*_min_ = 0.657, *T*_max_ = 0.746
50571 measured reflections
5002 independent reflections
4397 reflections with *I* > 2σ(*I*)
*R*_int_ = 0.051

#### Refinement

*R*[*F* ^2^ > 2σ(*F* ^2^)] = 0.041
*wR*(*F* ^2^) = 0.135
*S* = 0.98
5002 reflections
313 parameters
H-atom parameters constrained
Δρ_max_ = 1.91 e Å^−3^
Δρ_min_ = −1.12 e Å^−3^

### 

Data collection: *COLLECT* (Hooft, 1998 ▶); cell refinement: *SCALEPACK* (Otwinowski & Minor, 1997 ▶); data reduction: *DENZO* (Otwinowski & Minor, 1997 ▶) and *SCALEPACK*; program(s) used to solve structure: *SIR2002* (Burla *et al.*, 2003 ▶); program(s) used to refine structure: *SHELXL97* (Sheldrick, 2008*b* ▶); molecular graphics: *WinGX* (Farrugia, 1999 ▶); software used to prepare material for publication: *publCIF* (Westrip, 2010 ▶).

## Supplementary Material

Crystal structure: contains datablocks I, global. DOI: 10.1107/S1600536810017861/cv2705sup1.cif

Structure factors: contains datablocks I. DOI: 10.1107/S1600536810017861/cv2705Isup2.hkl

Additional supplementary materials:  crystallographic information; 3D view; checkCIF report

## Figures and Tables

**Table 1:** Hydrogen-bond geometry (Å, °)

*D*—H⋯*A*	*D*—H	H⋯*A*	*D*⋯*A*	*D*—H⋯*A*
C12—H12*C*⋯O2	0.98	2.57	3.473 (6)	153
C15—H15*B*⋯O1	0.98	2.47	3.349 (6)	149
C20—H20*B*⋯O1	0.98	2.60	3.329 (6)	132
C24—H24*C*⋯O2	0.98	2.52	3.432 (6)	155
C3—H3⋯O2^i^	0.95	2.51	3.284 (6)	139

Symmetry code: (i) 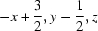.

**Fig. 1.**
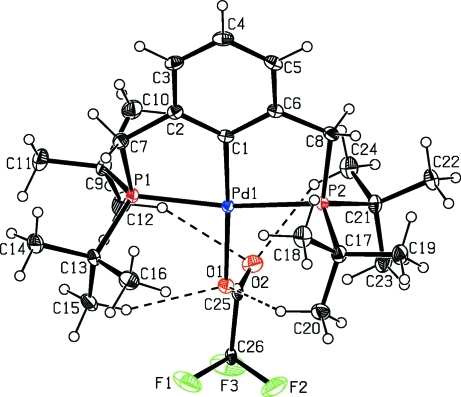
A molecular structure of (I), with the atom-numbering scheme. Displacement ellipsoids are drawn at the 30 % probability level. Intramolecular hydrogen bonds are shown by dashed lines.

**Fig. 2.**
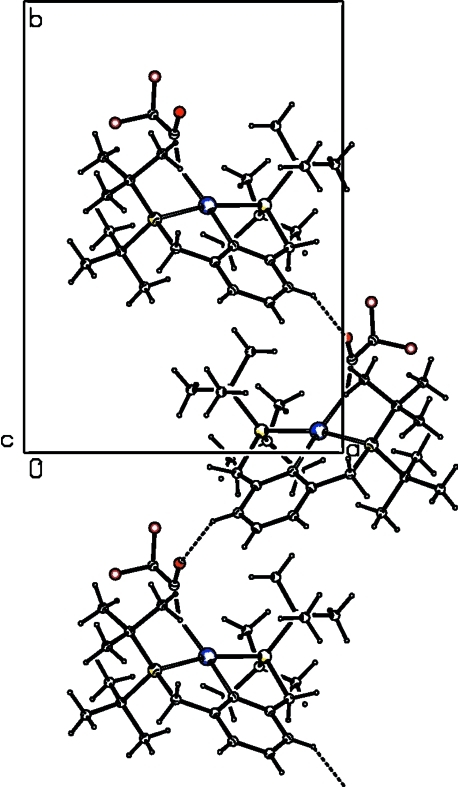
A crystal packing diagram of (I), viewed down the c axis, showing hydrogen-bonded chains of molecules running parallel to the b axis. Hydrogen bonds are indicated by dashed lines.

## supplementary crystallographic information

### Comment

The title compound (I) was prepared by cyclometallation of 1,3-[bis(ditertbutylphosphino)methyl]benzene (Johansson *et al.*, 2005). Our research has been directed towards carbon dioxide insertion into a series of compounds where compound (I) acts as a starting material for their synthesis (Johansson *et al.*, 2005; Johansson & Wendt, 2007).

The palladium atom in compound (I) exhibits a distorted square-planar geometry with the phosphorus atom coordinated trans to each other with a P1—Pd—P2 angle of 166.82 (4)° (Fig. 1). Coordination of the (PCP)-tridentate ligand leads to the formation of two five-membered rings. The Pd—O1 bond of 2.143 (3) Å is slightly elongated compared to its palladium acetate analogue with a Pd—O1 distance of 2.128 (2) Å (Johansson *et al.* 2005). This is as expected for a more electron deficient ligand. The Pd—P distances, Pd—P1 = 2.305 (1) Å and Pd—P2 = 2.317 (1) Å do not differ significantly. The P1—C2—C6—P2 torsion angle of +/-10.9 (3)° is similar to other PCPPd complexes. There are four moderate C–H···O intramolecular hydrogen bonds (Table 1). The angle between the least-squares planes through the trifluoroacetate atoms O1,O2,C25 and C26 and the phenyl ring is 73.5 (2)°. The deviation from the perpendicular orientation is caused by the C3–H3···O2*^i^* hydrogen bond (Fig. 2; Table 1), which self-assembles the molecules of (I) into infinite chains parallel to the *b* axis.

### Experimental

The title compound was prepared according to a literature procedure (Johansson *et al.*, 2005).

### Refinement

All hydrogen atoms were included in calculated positions as riding atoms, with C—H = 0.95Å for aromatic hydrogen atoms, 0.98Å for methyl hydrogen atoms and 0.99Å for methylene hydrogen atoms. The hydrogen atom U_iso_ parameters were set at 1.5U_eq_ for the methyl hydrogen atoms, and 1.2U_eq_ for all other hydrogen atoms.

Because of the bias in the data the C25 atom could not be refined anisotropically satisfactorily. Therefore, EADP constraint was applied in the refinement equallizing C25 with C26 atom. This results in high electronic density of 1.912 e Å^-3^ near C25 probably indicating some disorder or partial occupancy of another atom.

### Crystal data

**Table:**

[Pd(C_2_F_3_O_2_)(C_24_H_43_P_2_)]	*F*(000) = 2544
*M**_r_* = 612.94	*D*_x_ = 1.433 Mg m^−^^3^
Orthorhombic, *P**b**c**a*	Mo *K*α radiation, λ = 0.71073 Å
Hall symbol: -P 2ac 2ab	Cell parameters from 7600 reflections
*a* = 11.1239 (1) Å	θ = 0.4–28.3°
*b* = 15.7484 (2) Å	µ = 0.81 mm^−^^1^
*c* = 32.4400 (4) Å	*T* = 123 K
*V* = 5682.96 (11) Å^3^	Prism, pale brown
*Z* = 8	0.2 × 0.1 × 0.1 mm

### Data collection

**Table:**

Bruker–Nonius Kappa APEXII diffractometer	5002 independent reflections
Radiation source: Enraf–Nonius FR590	4397 reflections with *I* > 2σ(*I*)
horizonally mounted graphite crystal	*R*_int_ = 0.051
Detector resolution: 9 pixels mm^-1^	θ_max_ = 25.0°, θ_min_ = 1.3°
CCD rotation images, thick slices scans	*h* = −13→13
Absorption correction: multi-scan (*SADABS*; Sheldrick, 2008a)	*k* = −18→18
*T*_min_ = 0.657, *T*_max_ = 0.746	*l* = −38→38
50571 measured reflections	

### Refinement

**Table:**

Refinement on *F*^2^	Primary atom site location: structure-invariant direct methods
Least-squares matrix: full	Secondary atom site location: difference Fourier map
*R*[*F*^2^ > 2σ(*F*^2^)] = 0.041	Hydrogen site location: inferred from neighbouring sites
*wR*(*F*^2^) = 0.135	H-atom parameters constrained
*S* = 0.98	*w* = 1/[σ^2^(*F*_o_^2^) + (0.082*P*)^2^ + 22.4388*P*] where *P* = (*F*_o_^2^ + 2*F*_c_^2^)/3
5002 reflections	(Δ/σ)_max_ < 0.001
313 parameters	Δρ_max_ = 1.91 e Å^−^^3^
0 restraints	Δρ_min_ = −1.12 e Å^−^^3^

### Special details

**Table:**

Geometry. All s.u.'s (except the s.u. in the dihedral angle between two l.s. planes) are estimated using the full covariance matrix. The cell s.u.'s are taken into account individually in the estimation of s.u.'s in distances, angles and torsion angles; correlations between s.u.'s in cell parameters are only used when they are defined by crystal symmetry. An approximate (isotropic) treatment of cell s.u.'s is used for estimating s.u.'s involving l.s. planes.
Refinement. Refinement of *F*^2^ against ALL reflections. The weighted *R*-factor wR and goodness of fit *S* are based on *F*^2^, conventional *R*-factors *R* are based on *F*, with *F* set to zero for negative *F*^2^. The threshold expression of *F*^2^ > 2σ(*F*^2^) is used only for calculating *R*-factors(gt) etc. and is not relevant to the choice of reflections for refinement. *R*-factors based on *F*^2^ are statistically about twice as large as those based on *F*, and *R*- factors based on ALL data will be even larger.

### Fractional atomic coordinates and isotropic or equivalent isotropic displacement parameters (Å^2^)

**Table:**

	*x*	*y*	*z*	*U*_iso_*/*U*_eq_	
Pd1	0.92472 (3)	0.047957 (19)	0.129692 (9)	0.01622 (14)	
P1	0.74524 (10)	0.04831 (7)	0.09415 (3)	0.0196 (2)	
P2	1.08972 (9)	0.01753 (7)	0.17068 (3)	0.0175 (2)	
F1	1.0573 (4)	0.2240 (2)	0.01869 (10)	0.0683 (12)	
F2	1.2140 (3)	0.2306 (3)	0.05453 (13)	0.0704 (11)	
F3	1.0899 (4)	0.3333 (2)	0.05529 (12)	0.0714 (12)	
O1	1.0127 (3)	0.13118 (19)	0.08700 (9)	0.0259 (7)	
O2	1.0118 (3)	0.2545 (2)	0.12135 (10)	0.0372 (8)	
C1	0.8431 (4)	−0.0415 (2)	0.16433 (12)	0.0183 (8)	
C2	0.7283 (4)	−0.0729 (3)	0.15436 (12)	0.0211 (8)	
C3	0.6719 (4)	−0.1335 (3)	0.17900 (13)	0.0255 (9)	
H3	0.5928	−0.1518	0.1725	0.031*	
C4	0.7300 (4)	−0.1670 (3)	0.21277 (13)	0.0284 (10)	
H4	0.6916	−0.2087	0.2293	0.034*	
C5	0.8446 (4)	−0.1395 (3)	0.22229 (13)	0.0261 (9)	
H5	0.8854	−0.1635	0.2452	0.031*	
C6	0.9013 (4)	−0.0772 (3)	0.19889 (12)	0.0198 (8)	
C7	0.6679 (4)	−0.0456 (3)	0.11482 (14)	0.0259 (10)	
H7A	0.5824	−0.0320	0.1202	0.031*	
H7B	0.6710	−0.0924	0.0945	0.031*	
C8	1.0236 (4)	−0.0473 (3)	0.21179 (13)	0.0242 (9)	
H8A	1.0759	−0.0968	0.2175	0.029*	
H8B	1.0171	−0.0133	0.2374	0.029*	
C9	0.6442 (4)	0.1402 (3)	0.10593 (14)	0.0265 (9)	
C10	0.6190 (5)	0.1345 (3)	0.15268 (16)	0.0390 (12)	
H10A	0.5734	0.1846	0.1615	0.058*	
H10B	0.6953	0.1321	0.1677	0.058*	
H10C	0.5721	0.0832	0.1585	0.058*	
C11	0.5250 (4)	0.1391 (3)	0.08229 (16)	0.0387 (12)	
H11A	0.4871	0.0833	0.0853	0.058*	
H11B	0.5402	0.1505	0.0531	0.058*	
H11C	0.4715	0.1828	0.0935	0.058*	
C12	0.7101 (4)	0.2240 (3)	0.09810 (18)	0.0382 (12)	
H12A	0.6598	0.2714	0.1073	0.057*	
H12B	0.7265	0.2299	0.0686	0.057*	
H12C	0.7861	0.2243	0.1134	0.057*	
C13	0.7625 (4)	0.0257 (3)	0.03776 (13)	0.0269 (9)	
C14	0.6481 (5)	−0.0096 (4)	0.01783 (15)	0.0397 (12)	
H14A	0.5867	0.0349	0.0169	0.060*	
H14B	0.6184	−0.0576	0.0341	0.060*	
H14C	0.6662	−0.0287	−0.0102	0.060*	
C15	0.8009 (5)	0.1061 (3)	0.01466 (14)	0.0359 (11)	
H15A	0.8214	0.0915	−0.0139	0.054*	
H15B	0.8711	0.1311	0.0283	0.054*	
H15C	0.7346	0.1471	0.0148	0.054*	
C16	0.8633 (5)	−0.0405 (3)	0.03433 (16)	0.0375 (12)	
H16A	0.8414	−0.0912	0.0502	0.056*	
H16B	0.9381	−0.0166	0.0453	0.056*	
H16C	0.8748	−0.0559	0.0053	0.056*	
C17	1.1956 (4)	−0.0536 (3)	0.14181 (14)	0.0256 (9)	
C18	1.1218 (5)	−0.1331 (3)	0.13018 (15)	0.0336 (11)	
H18A	1.1705	−0.1704	0.1126	0.050*	
H18B	1.0493	−0.1158	0.1152	0.050*	
H18C	1.0988	−0.1636	0.1553	0.050*	
C19	1.3059 (4)	−0.0812 (3)	0.16619 (15)	0.0346 (11)	
H19A	1.3526	−0.1220	0.1499	0.052*	
H19B	1.2804	−0.1080	0.1920	0.052*	
H19C	1.3558	−0.0315	0.1724	0.052*	
C20	1.2322 (4)	−0.0091 (3)	0.10201 (14)	0.0337 (11)	
H20A	1.2743	0.0439	0.1086	0.050*	
H20B	1.1603	0.0036	0.0857	0.050*	
H20C	1.2857	−0.0462	0.0861	0.050*	
C21	1.1686 (4)	0.1037 (3)	0.20004 (13)	0.0251 (9)	
C22	1.2397 (5)	0.0707 (3)	0.23723 (14)	0.0337 (11)	
H22A	1.3060	0.0348	0.2276	0.050*	
H22B	1.1864	0.0373	0.2550	0.050*	
H22C	1.2720	0.1188	0.2528	0.050*	
C23	1.2516 (4)	0.1555 (3)	0.17256 (15)	0.0359 (11)	
H23A	1.2772	0.2068	0.1873	0.054*	
H23B	1.2088	0.1717	0.1474	0.054*	
H23C	1.3223	0.1215	0.1654	0.054*	
C24	1.0671 (4)	0.1621 (3)	0.21528 (16)	0.0348 (11)	
H24A	1.1013	0.2078	0.2321	0.052*	
H24B	1.0102	0.1291	0.2319	0.052*	
H24C	1.0251	0.1867	0.1915	0.052*	
C25	1.0297 (4)	0.2060 (2)	0.09397 (12)	0.0189 (6)	
C26	1.0960 (3)	0.2495 (2)	0.05532 (12)	0.0189 (6)	

### Atomic displacement parameters (Å^2^)

**Table:**

	*U*^11^	*U*^22^	*U*^33^	*U*^12^	*U*^13^	*U*^23^
Pd1	0.0148 (2)	0.0174 (2)	0.0165 (2)	−0.00160 (11)	−0.00134 (11)	0.00210 (11)
P1	0.0166 (5)	0.0229 (5)	0.0194 (5)	−0.0025 (4)	−0.0033 (4)	0.0014 (4)
P2	0.0160 (5)	0.0193 (5)	0.0172 (5)	0.0007 (4)	−0.0017 (4)	0.0004 (4)
F1	0.103 (3)	0.074 (3)	0.0280 (17)	−0.052 (2)	0.0003 (17)	0.0064 (16)
F2	0.0307 (17)	0.098 (3)	0.083 (3)	−0.0020 (18)	0.0170 (17)	0.039 (2)
F3	0.120 (3)	0.0301 (17)	0.064 (2)	−0.0164 (19)	0.031 (2)	0.0090 (16)
O1	0.0257 (16)	0.0282 (17)	0.0237 (15)	−0.0023 (13)	−0.0002 (12)	0.0036 (13)
O2	0.036 (2)	0.038 (2)	0.0371 (19)	−0.0042 (15)	0.0066 (14)	−0.0007 (16)
C1	0.024 (2)	0.0131 (18)	0.0175 (19)	−0.0003 (15)	0.0041 (16)	−0.0008 (15)
C2	0.022 (2)	0.0194 (19)	0.022 (2)	−0.0006 (17)	0.0021 (16)	−0.0042 (17)
C3	0.027 (2)	0.023 (2)	0.027 (2)	−0.0064 (18)	0.0076 (18)	−0.0031 (18)
C4	0.036 (3)	0.020 (2)	0.029 (2)	−0.0036 (19)	0.0108 (19)	−0.0002 (18)
C5	0.035 (2)	0.024 (2)	0.019 (2)	0.0034 (19)	0.0039 (18)	0.0033 (17)
C6	0.024 (2)	0.0164 (19)	0.019 (2)	0.0013 (17)	0.0028 (16)	−0.0003 (16)
C7	0.025 (2)	0.026 (2)	0.026 (2)	−0.0074 (17)	−0.0013 (19)	0.0024 (17)
C8	0.026 (2)	0.028 (2)	0.018 (2)	0.0027 (18)	0.0005 (17)	0.0053 (17)
C9	0.019 (2)	0.027 (2)	0.034 (2)	0.0013 (18)	−0.0010 (18)	−0.0007 (19)
C10	0.032 (3)	0.044 (3)	0.041 (3)	0.008 (2)	0.006 (2)	−0.006 (2)
C11	0.025 (2)	0.046 (3)	0.046 (3)	0.006 (2)	−0.008 (2)	0.000 (2)
C12	0.031 (3)	0.025 (2)	0.059 (3)	0.001 (2)	−0.005 (2)	0.001 (2)
C13	0.025 (2)	0.037 (2)	0.019 (2)	−0.0044 (19)	−0.0059 (17)	−0.0008 (18)
C14	0.037 (3)	0.055 (3)	0.027 (2)	−0.015 (2)	−0.008 (2)	−0.005 (2)
C15	0.040 (3)	0.048 (3)	0.019 (2)	−0.012 (2)	−0.006 (2)	0.005 (2)
C16	0.041 (3)	0.042 (3)	0.029 (3)	0.002 (2)	0.003 (2)	−0.013 (2)
C17	0.023 (2)	0.029 (2)	0.026 (2)	0.0079 (18)	−0.0017 (18)	−0.0046 (18)
C18	0.035 (3)	0.026 (2)	0.039 (3)	0.009 (2)	−0.004 (2)	−0.012 (2)
C19	0.026 (2)	0.044 (3)	0.034 (3)	0.014 (2)	−0.004 (2)	−0.004 (2)
C20	0.032 (3)	0.044 (3)	0.026 (2)	0.008 (2)	0.0050 (19)	−0.001 (2)
C21	0.025 (2)	0.024 (2)	0.027 (2)	−0.0019 (17)	−0.0077 (18)	−0.0021 (18)
C22	0.036 (3)	0.037 (3)	0.028 (2)	−0.001 (2)	−0.012 (2)	−0.004 (2)
C23	0.030 (2)	0.035 (3)	0.043 (3)	−0.011 (2)	−0.008 (2)	0.003 (2)
C24	0.040 (3)	0.027 (2)	0.038 (3)	0.002 (2)	−0.008 (2)	−0.010 (2)
C25	0.0206 (14)	0.0136 (14)	0.0227 (15)	−0.0055 (11)	−0.0091 (12)	0.0003 (12)
C26	0.0206 (14)	0.0136 (14)	0.0227 (15)	−0.0055 (11)	−0.0091 (12)	0.0003 (12)

### Geometric parameters (Å, °)

**Table:**

Pd1—C1	2.018 (4)	C12—H12B	0.9800
Pd1—O1	2.143 (3)	C12—H12C	0.9800
Pd1—P1	2.3054 (11)	C13—C14	1.532 (6)
Pd1—P2	2.3165 (10)	C13—C15	1.531 (6)
P1—C7	1.838 (4)	C13—C16	1.535 (7)
P1—C9	1.871 (4)	C14—H14A	0.9800
P1—C13	1.874 (4)	C14—H14B	0.9800
P2—C8	1.833 (4)	C14—H14C	0.9800
P2—C17	1.876 (4)	C15—H15A	0.9800
P2—C21	1.876 (4)	C15—H15B	0.9800
F1—C26	1.326 (5)	C15—H15C	0.9800
F2—C26	1.346 (5)	C16—H16A	0.9800
F3—C26	1.323 (5)	C16—H16B	0.9800
O1—C25	1.215 (5)	C16—H16C	0.9800
O2—C25	1.188 (5)	C17—C19	1.523 (6)
C1—C2	1.407 (6)	C17—C20	1.524 (6)
C1—C6	1.411 (6)	C17—C18	1.545 (6)
C2—C3	1.394 (6)	C18—H18A	0.9800
C2—C7	1.510 (6)	C18—H18B	0.9800
C3—C4	1.377 (6)	C18—H18C	0.9800
C3—H3	0.9500	C19—H19A	0.9800
C4—C5	1.381 (7)	C19—H19B	0.9800
C4—H4	0.9500	C19—H19C	0.9800
C5—C6	1.392 (6)	C20—H20A	0.9800
C5—H5	0.9500	C20—H20B	0.9800
C6—C8	1.499 (6)	C20—H20C	0.9800
C7—H7A	0.9900	C21—C23	1.521 (6)
C7—H7B	0.9900	C21—C22	1.533 (6)
C8—H8A	0.9900	C21—C24	1.538 (6)
C8—H8B	0.9900	C22—H22A	0.9800
C9—C12	1.531 (6)	C22—H22B	0.9800
C9—C11	1.532 (6)	C22—H22C	0.9800
C9—C10	1.545 (7)	C23—H23A	0.9800
C10—H10A	0.9800	C23—H23B	0.9800
C10—H10B	0.9800	C23—H23C	0.9800
C10—H10C	0.9800	C24—H24A	0.9800
C11—H11A	0.9800	C24—H24B	0.9800
C11—H11B	0.9800	C24—H24C	0.9800
C11—H11C	0.9800	C25—C26	1.607 (6)
C12—H12A	0.9800		
			
C1—Pd1—O1	172.85 (13)	C15—C13—P1	110.5 (3)
C1—Pd1—P1	83.71 (12)	C16—C13—P1	105.9 (3)
O1—Pd1—P1	94.06 (8)	C13—C14—H14A	109.5
C1—Pd1—P2	83.83 (12)	C13—C14—H14B	109.5
O1—Pd1—P2	97.79 (8)	H14A—C14—H14B	109.5
P1—Pd1—P2	166.82 (4)	C13—C14—H14C	109.5
C7—P1—C9	105.5 (2)	H14A—C14—H14C	109.5
C7—P1—C13	104.6 (2)	H14B—C14—H14C	109.5
C9—P1—C13	114.1 (2)	C13—C15—H15A	109.5
C7—P1—Pd1	102.78 (15)	C13—C15—H15B	109.5
C9—P1—Pd1	114.83 (14)	H15A—C15—H15B	109.5
C13—P1—Pd1	113.51 (15)	C13—C15—H15C	109.5
C8—P2—C17	106.4 (2)	H15A—C15—H15C	109.5
C8—P2—C21	102.8 (2)	H15B—C15—H15C	109.5
C17—P2—C21	113.0 (2)	C13—C16—H16A	109.5
C8—P2—Pd1	102.39 (14)	C13—C16—H16B	109.5
C17—P2—Pd1	109.55 (15)	H16A—C16—H16B	109.5
C21—P2—Pd1	120.84 (14)	C13—C16—H16C	109.5
C25—O1—Pd1	123.0 (3)	H16A—C16—H16C	109.5
C2—C1—C6	117.4 (4)	H16B—C16—H16C	109.5
C2—C1—Pd1	121.7 (3)	C19—C17—C20	110.9 (4)
C6—C1—Pd1	120.9 (3)	C19—C17—C18	108.8 (4)
C3—C2—C1	121.1 (4)	C20—C17—C18	107.9 (4)
C3—C2—C7	118.8 (4)	C19—C17—P2	114.7 (3)
C1—C2—C7	120.0 (4)	C20—C17—P2	108.5 (3)
C4—C3—C2	120.5 (4)	C18—C17—P2	105.8 (3)
C4—C3—H3	119.7	C17—C18—H18A	109.5
C2—C3—H3	119.7	C17—C18—H18B	109.5
C3—C4—C5	119.4 (4)	H18A—C18—H18B	109.5
C3—C4—H4	120.3	C17—C18—H18C	109.5
C5—C4—H4	120.3	H18A—C18—H18C	109.5
C4—C5—C6	121.2 (4)	H18B—C18—H18C	109.5
C4—C5—H5	119.4	C17—C19—H19A	109.5
C6—C5—H5	119.4	C17—C19—H19B	109.5
C5—C6—C1	120.4 (4)	H19A—C19—H19B	109.5
C5—C6—C8	118.7 (4)	C17—C19—H19C	109.5
C1—C6—C8	120.9 (4)	H19A—C19—H19C	109.5
C2—C7—P1	109.3 (3)	H19B—C19—H19C	109.5
C2—C7—H7A	109.8	C17—C20—H20A	109.5
P1—C7—H7A	109.8	C17—C20—H20B	109.5
C2—C7—H7B	109.8	H20A—C20—H20B	109.5
P1—C7—H7B	109.8	C17—C20—H20C	109.5
H7A—C7—H7B	108.3	H20A—C20—H20C	109.5
C6—C8—P2	109.6 (3)	H20B—C20—H20C	109.5
C6—C8—H8A	109.7	C23—C21—C22	109.3 (4)
P2—C8—H8A	109.7	C23—C21—C24	108.2 (4)
C6—C8—H8B	109.7	C22—C21—C24	109.2 (4)
P2—C8—H8B	109.7	C23—C21—P2	112.0 (3)
H8A—C8—H8B	108.2	C22—C21—P2	113.3 (3)
C12—C9—C11	109.9 (4)	C24—C21—P2	104.6 (3)
C12—C9—C10	107.4 (4)	C21—C22—H22A	109.5
C11—C9—C10	109.5 (4)	C21—C22—H22B	109.5
C12—C9—P1	110.2 (3)	H22A—C22—H22B	109.5
C11—C9—P1	114.2 (3)	C21—C22—H22C	109.5
C10—C9—P1	105.4 (3)	H22A—C22—H22C	109.5
C9—C10—H10A	109.5	H22B—C22—H22C	109.5
C9—C10—H10B	109.5	C21—C23—H23A	109.5
H10A—C10—H10B	109.5	C21—C23—H23B	109.5
C9—C10—H10C	109.5	H23A—C23—H23B	109.5
H10A—C10—H10C	109.5	C21—C23—H23C	109.5
H10B—C10—H10C	109.5	H23A—C23—H23C	109.5
C9—C11—H11A	109.5	H23B—C23—H23C	109.5
C9—C11—H11B	109.5	C21—C24—H24A	109.5
H11A—C11—H11B	109.5	C21—C24—H24B	109.5
C9—C11—H11C	109.5	H24A—C24—H24B	109.5
H11A—C11—H11C	109.5	C21—C24—H24C	109.5
H11B—C11—H11C	109.5	H24A—C24—H24C	109.5
C9—C12—H12A	109.5	H24B—C24—H24C	109.5
C9—C12—H12B	109.5	O2—C25—O1	137.4 (4)
H12A—C12—H12B	109.5	O2—C25—C26	112.8 (3)
C9—C12—H12C	109.5	O1—C25—C26	109.8 (3)
H12A—C12—H12C	109.5	F3—C26—F1	106.6 (4)
H12B—C12—H12C	109.5	F3—C26—F2	105.6 (4)
C14—C13—C15	109.0 (4)	F1—C26—F2	103.4 (4)
C14—C13—C16	109.2 (4)	F3—C26—C25	113.7 (4)
C15—C13—C16	108.8 (4)	F1—C26—C25	114.9 (3)
C14—C13—P1	113.3 (3)	F2—C26—C25	111.6 (3)
			
C1—Pd1—P1—C7	−11.03 (19)	C21—P2—C8—C6	141.5 (3)
O1—Pd1—P1—C7	162.15 (17)	Pd1—P2—C8—C6	15.5 (3)
P2—Pd1—P1—C7	8.1 (2)	C7—P1—C9—C12	168.5 (3)
C1—Pd1—P1—C9	102.93 (19)	C13—P1—C9—C12	−77.3 (4)
O1—Pd1—P1—C9	−83.89 (18)	Pd1—P1—C9—C12	56.2 (4)
P2—Pd1—P1—C9	122.0 (2)	C7—P1—C9—C11	−67.2 (4)
C1—Pd1—P1—C13	−123.4 (2)	C13—P1—C9—C11	46.9 (4)
O1—Pd1—P1—C13	49.83 (18)	Pd1—P1—C9—C11	−179.6 (3)
P2—Pd1—P1—C13	−104.2 (2)	C7—P1—C9—C10	52.9 (3)
C1—Pd1—P2—C8	−11.61 (18)	C13—P1—C9—C10	167.1 (3)
O1—Pd1—P2—C8	175.40 (17)	Pd1—P1—C9—C10	−59.5 (3)
P1—Pd1—P2—C8	−30.7 (2)	C7—P1—C13—C14	45.2 (4)
C1—Pd1—P2—C17	101.06 (19)	C9—P1—C13—C14	−69.5 (4)
O1—Pd1—P2—C17	−71.92 (18)	Pd1—P1—C13—C14	156.4 (3)
P1—Pd1—P2—C17	82.0 (2)	C7—P1—C13—C15	167.7 (3)
C1—Pd1—P2—C21	−124.9 (2)	C9—P1—C13—C15	53.1 (4)
O1—Pd1—P2—C21	62.15 (19)	Pd1—P1—C13—C15	−81.0 (3)
P1—Pd1—P2—C21	−144.0 (2)	C7—P1—C13—C16	−74.6 (3)
P1—Pd1—O1—C25	102.9 (3)	C9—P1—C13—C16	170.7 (3)
P2—Pd1—O1—C25	−82.9 (3)	Pd1—P1—C13—C16	36.7 (3)
P1—Pd1—C1—C2	4.9 (3)	C8—P2—C17—C19	−67.6 (4)
P2—Pd1—C1—C2	−170.8 (3)	C21—P2—C17—C19	44.4 (4)
P1—Pd1—C1—C6	−177.3 (3)	Pd1—P2—C17—C19	−177.6 (3)
P2—Pd1—C1—C6	7.0 (3)	C8—P2—C17—C20	167.9 (3)
C6—C1—C2—C3	3.3 (6)	C21—P2—C17—C20	−80.0 (4)
Pd1—C1—C2—C3	−178.7 (3)	Pd1—P2—C17—C20	57.9 (3)
C6—C1—C2—C7	−172.2 (4)	C8—P2—C17—C18	52.3 (3)
Pd1—C1—C2—C7	5.7 (5)	C21—P2—C17—C18	164.4 (3)
C1—C2—C3—C4	−3.0 (6)	Pd1—P2—C17—C18	−57.7 (3)
C7—C2—C3—C4	172.6 (4)	C8—P2—C21—C23	168.4 (3)
C2—C3—C4—C5	0.6 (6)	C17—P2—C21—C23	54.1 (4)
C3—C4—C5—C6	1.4 (6)	Pd1—P2—C21—C23	−78.5 (3)
C4—C5—C6—C1	−1.0 (6)	C8—P2—C21—C22	44.2 (4)
C4—C5—C6—C8	177.5 (4)	C17—P2—C21—C22	−70.1 (4)
C2—C1—C6—C5	−1.4 (6)	Pd1—P2—C21—C22	157.3 (3)
Pd1—C1—C6—C5	−179.3 (3)	C8—P2—C21—C24	−74.6 (3)
C2—C1—C6—C8	−179.8 (4)	C17—P2—C21—C24	171.1 (3)
Pd1—C1—C6—C8	2.3 (5)	Pd1—P2—C21—C24	38.5 (3)
C3—C2—C7—P1	168.9 (3)	Pd1—O1—C25—O2	1.7 (7)
C1—C2—C7—P1	−15.4 (5)	Pd1—O1—C25—C26	−179.2 (2)
C9—P1—C7—C2	−104.4 (3)	O2—C25—C26—F3	−17.5 (5)
C13—P1—C7—C2	135.1 (3)	O1—C25—C26—F3	163.2 (4)
Pd1—P1—C7—C2	16.3 (3)	O2—C25—C26—F1	−140.7 (4)
C5—C6—C8—P2	168.6 (3)	O1—C25—C26—F1	39.9 (5)
C1—C6—C8—P2	−13.0 (5)	O2—C25—C26—F2	101.9 (5)
C17—P2—C8—C6	−99.4 (3)	O1—C25—C26—F2	−77.4 (4)

### Hydrogen-bond geometry (Å, °)

**Table:**

*D*—H···*A*	*D*—H	H···*A*	*D*···*A*	*D*—H···*A*
C12—H12C···O2	0.98	2.57	3.473 (6)	153
C15—H15B···O1	0.98	2.47	3.349 (6)	149
C20—H20B···O1	0.98	2.60	3.329 (6)	132
C24—H24C···O2	0.98	2.52	3.432 (6)	155
C3—H3···O2^i^	0.95	2.51	3.284 (6)	139

Symmetry codes:  (i) −*x*+3/2, *y*−1/2, *z*.
